# Increased gut permeability in cancer cachexia: mechanisms and clinical relevance

**DOI:** 10.18632/oncotarget.24804

**Published:** 2018-04-06

**Authors:** Laure B. Bindels, Audrey M. Neyrinck, Audrey Loumaye, Emilie Catry, Hannah Walgrave, Claire Cherbuy, Sophie Leclercq, Matthias Van Hul, Hubert Plovier, Barbara Pachikian, Luis G. Bermúdez-Humarán, Philippe Langella, Patrice D. Cani, Jean-Paul Thissen, Nathalie M. Delzenne

**Affiliations:** ^1^ Metabolism and Nutrition Research Group, Louvain Drug Research Institute, Université Catholique de Louvain, Brussels, Belgium; ^2^ Endocrinology, Diabetology and Nutrition Department, Institut de Recherches Expérimentales et Cliniques, Université Catholique de Louvain, Cliniques Universitaires Saint-Luc, Brussels, Belgium; ^3^ Micalis Institute, INRA, AgroParisTech, Université Paris-Saclay, Jouy-en-Josas, France; ^4^ Pôle Clinique, Psychiatrie, Institute of Neuroscience, Université Catholique de Louvain, Brussels, Belgium; ^5^ Walloon Excellence in Life Sciences and BIOtechnology (WELBIO), Louvain Drug Research Institute, Université Catholique de Louvain, Brussels, Belgium

**Keywords:** cancer cachexia, gut barrier function, gut dysbiosis, lipopolysaccharide-binding protein, Enterobacteriaceae

## Abstract

Intestinal disorders often occur in cancer patients, in association with body weight loss, and this alteration is commonly attributed to the chemotherapy. Here, using a mouse model of cancer cachexia induced by ectopic transplantation of C26 cancer cells, we discovered a profound alteration in the gut functions (gut permeability, epithelial turnover, gut immunity, microbial dysbiosis) independently of any chemotherapy. These alterations occurred independently of anorexia and were driven by interleukin 6. Gut dysfunction was found to be resistant to treatments with an anti-inflammatory bacterium (*Faecalibacterium prausnitzii*) or with gut peptides involved in intestinal cell renewal (teduglutide, a glucagon-like peptide 2 analogue). The translational value of our findings was evaluated in 152 colorectal and lung cancer patients with or without cachexia. The serum level of the lipopolysaccharide-binding protein, often presented as a reflection of the bacterial antigen load, was not only increased in cachectic mice and cancer patients, but also strongly correlated with the serum IL-6 level and predictive of death and cachexia occurrence in these patients. Altogether, our data highlight profound alterations of the intestinal homeostasis in cancer cachexia occurring independently of any chemotherapy and food intake reduction, with potential relevance in humans. In addition, we point out the lipopolysaccharide-binding protein as a new biomarker of cancer cachexia related to gut dysbiosis.

## INTRODUCTION

Cachexia is a complex multi-organ syndrome characterized by body weight loss, weakness, muscle atrophy, fat depletion, anorexia and inflammation. Cachexia accompanies the terminal phase of many chronic diseases such as cancer and chronic heart failure [1]. Clinically, cachexia results in increased morbidity and mortality rates as well as reduced tolerance to anti-cancer treatments. It also complicates cancer patients’ management [1, 2]. Most anticancer treatments, including chemotherapy (e.g. oxaliplatin, cyclophosphamide, methotrexate), immunotherapy (e.g. anti-CTLA4) and radiotherapy, will directly or indirectly alter the gut barrier function, leading to diarrhea and nutrient malabsorption [3]. Such gastrointestinal side effects worsen cachexia [4]. Cancer cachexia prevalence was estimated at one million people in Europe in 2016 with about 90% of cancer patients being at risk of cachexia [1, 2]. Currently, only limited therapeutic options exist for this important medical challenge and new approaches to tackle this syndrome are needed [5, 6]. In this context, targeting the gut and its inhabitants (the gut microbiota) represents an exciting opportunity for this public health issue [3, 7, 8].

The gut microbiota is considered a crucial regulator of host immunity and metabolism and microbial dysbiosis has been associated with the occurrence and/or evolution of several metabolic and inflammatory diseases [9–13]. Links between gut microbiota and cancer have been studied for years [14, 15], but it is only recently that the existence of a crosstalk between gut microbiota and metabolic alterations -including cachexia- occurring during cancer has been proposed based on three main experimental observations. First, administration of lactobacilli counteracted muscle atrophy in mouse models of cancer cachexia [16, 17]. Second, a microbial signature was found in models of cancer cachexia, characterized by an increase in *Enterobacteriaceae* [18, 19]. *Enterobacteriaceae* are Gram-negative bacteria that activate pro-inflammatory processes through the binding of lipopolysaccharides (LPS) on Toll-like receptor 4 (TLR4). Third, nutritional interventions that target the microbiota (including prebiotics and/or probiotics) decreased cancer progression, reduced morbidity and fat mass loss, and increased survival of cachectic mice [18, 19].

Our latest study, performed in leukemic mice with cachexia without any anticancer therapy, showed changes in the ileal expression of key genes involved in the control of immunity, gut barrier and microbiota shaping [18], suggesting that cancer cell presence may trigger alterations of the gut barrier function independently of any anticancer intervention. Several factors, including epithelium renewal, presence of immune cells, secretion of glycoproteins such as mucins, expression of tight junction proteins, release of agents that prevent the translocation of gut microbes (e.g. immunoglobulin A), and secretion of antimicrobial proteins participate to the gut barrier function that protects the host against the translocation of microbial compounds [20]. An altered gut barrier may be accompanied by an increased translocation of pro-inflammatory microbial antigens reaching the liver and other peripheral organs, as described in obesity-related metabolic disorders [10]. Exogenous antigen load in the host is often reflected in the levels of the lipopolysaccharide binding protein (LBP), an acute phase response protein [21, 22].

These studies raised several questions: Do the gut microbiota and the intestine play a role in cancer cachexia, independently of any anticancer treatment? What are the mechanisms involved in this crosstalk? To answer these questions, we used biochemical, morphological and molecular profiling of the host as well as next-generation sequencing of the bacterial microbiome to gain in-depth insight into the impact of cancer and cachexia on the gut barrier function and microbiota, and to identify pathways that are involved in these gut microbiota-host interactions. Using these approaches, we identified one cytokine, interleukin-6 (IL-6), as a driver, not only of cachexia, but also of gut barrier alterations and microbial dysbiosis in a preclinical model of cancer cachexia. In line with this altered gut barrier function, LBP and IL-6 levels were increased in cancer cachectic patients versus cancer non-cachectic patients in two populations of patients (lung cancer and colorectal cancer). Furthermore, we identified the LBP level as a predictive factor of survival and of several cachectic hallmarks.

## RESULTS

### The gut-liver homeostasis and microbial ecosystem are disrupted in the C26 mouse model of cancer cachexia

**Figure 1:**
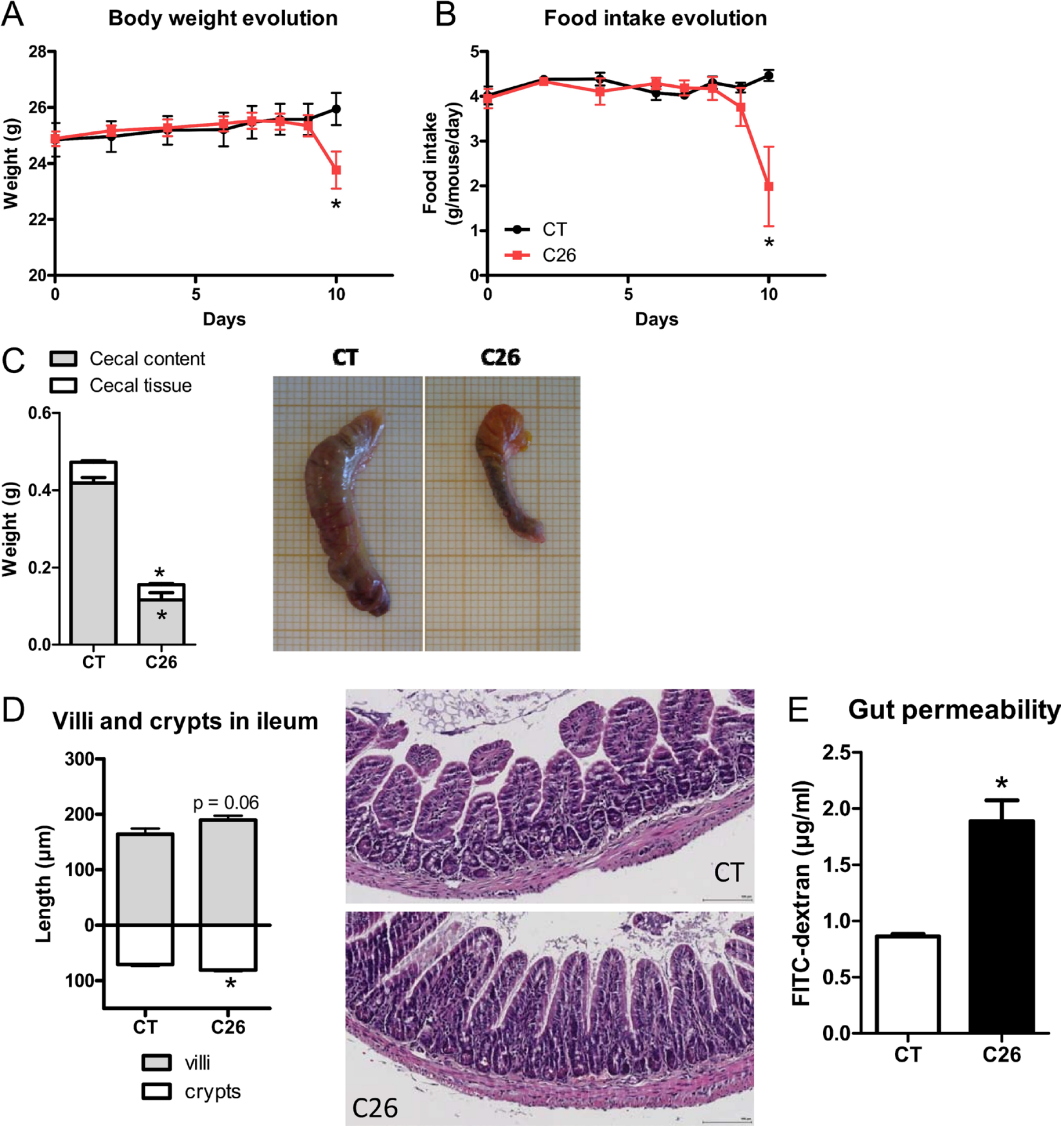
Impact of cancer cachexia on the gastrointestinal tract (**A**–**B**) Body weight and food intake evolution. (**C**) Cecal content and tissue weight with representative pictures. (**D**) Villi length and crypt depth in ileum with representative photomicrographs of hematoxylin-eosin stained tissues. (**E**) Gut permeability as assessed *in vivo* using FITC-dextran. Mice received either a sham-injection (CT) or an injection with cancer cells (C26). *n* = 7–8, ^*^p < 0.05.

**Figure 2:**
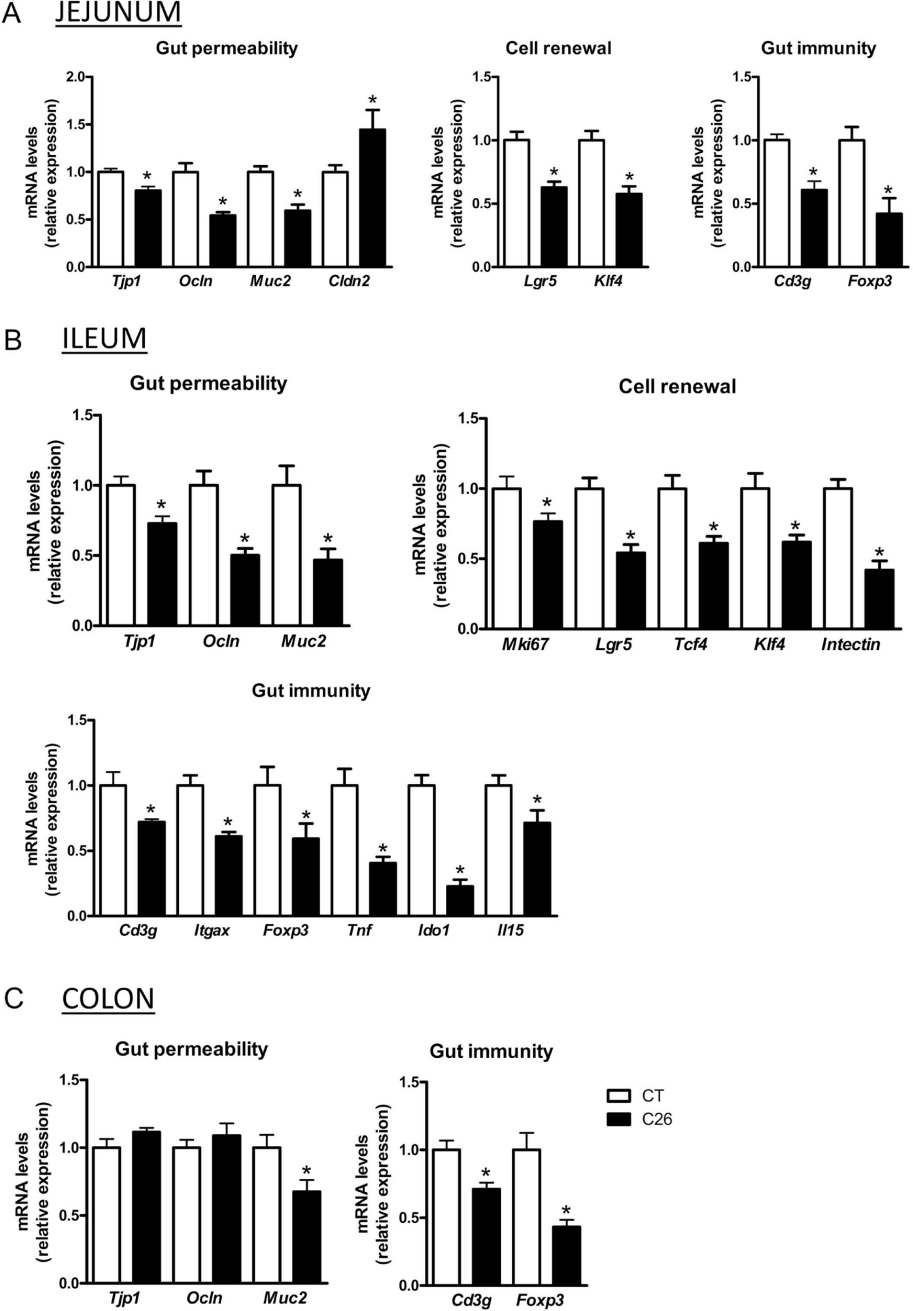
Gut integrity, cell renewal and gut immunity biomarkers are decreased in cachectic mice mRNA expression of markers involved in gut barrier integrity (*Tjp1, Ocln, Muc2, Cldn2*), proliferation, shedding and differentiation of epithelial and specialized cell lineages (*Mki67, Lgr5, Tcf4, Klf4, intectin*) and gut immunity (*Cd3g, Itgax, Foxp3, Tnf, Ido1, Il15*), in the jejunum (**A**), ileum (**B**) and colon (**C**) of sham-injected (CT) and cachectic mice (C26). *n* = 7–8, ^*^p < 0.05.

**Figure 3:**
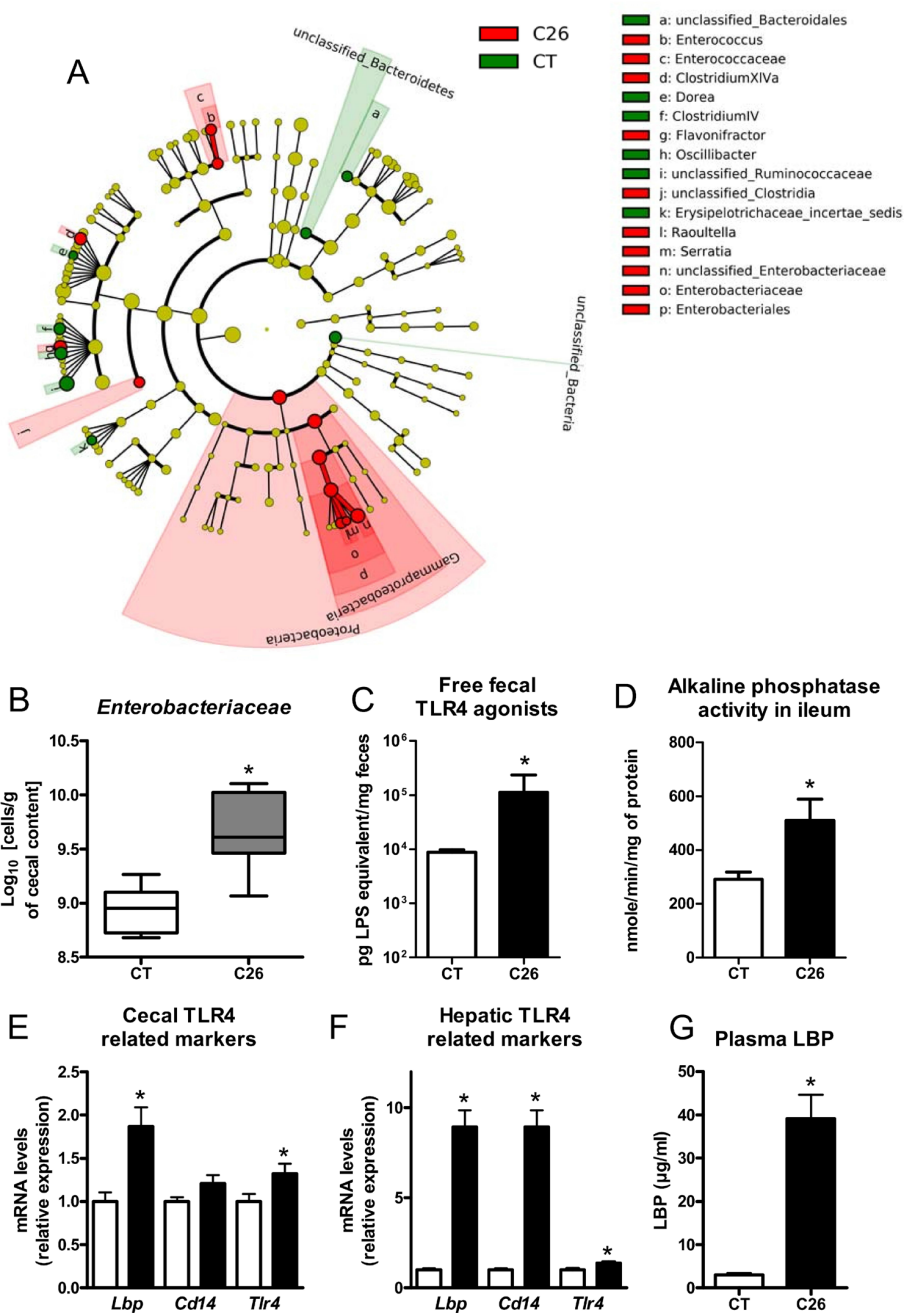
Gut microbial changes and increased expression of markers related to the TLR4 pathway (**A**) LEfSe cladogram in red for the taxa enriched in cachectic mice (C26) and in green for the taxa enriched in sham-injected mice (CT). (**B**) Cecal *Enterobacteriaceae* levels, as determined by qPCR. (**C**) Fecal activity in free TLR4 agonists. (**D**) Alkaline phosphatase activity. (**E**–**F**) mRNA expression of markers related to the TLR4 pathway in the cecal tissue and liver. (**G**) Plasma LBP levels. *n* = 7–8, ^*^p < 0.05.

The C26 cachexia model consists of a subcutaneous injection of colon carcinoma cells [23]. This model is characterized by a relatively small tumor mass as well as a decreased food intake and loss of body weight, due to muscle atrophy and later on to adipose tissue loss (Figure 1, Supplementary Figure 1). We found several indications of altered intestinal homeostasis in cachectic mice. Cecal content and tissue weight were decreased, villi length and crypt depth were increased and gut permeability assessed *in vivo* was raised by two-fold (Figure 1). Moreover, the expression of several markers of gut barrier function, cell renewal and gut immunity was diminished, mainly in the ileum but also in the jejunum and in the colon (Figure 2). The expression of claudin 2, a channel-forming protein, was increased in the jejunum of cachectic mice. The microbiota of cachectic mice showed an altered composition, with an increase in *Enterobacteriaceae*, as previously documented [18] (Figure 3A and 3B). Accordingly, the fecal content in free TLR4 agonists was increased in cachectic mice (Figure 3C). The activity of the intestinal alkaline phosphatase (an enzyme induced by the TLR4 agonists lipopolysaccharides (LPS) and involved in LPS detoxification [24]) was enhanced in cachectic mice (Figure 3D). Markers of the TLR4 pathway were increased in the cecal and liver tissues (Figure 3E and 3F). In accordance with these findings and the increased gut permeability, plasma LBP levels were increased in cachectic mice (Figure 3G). Gut permeability, as assessed by FITC-dextran, was strongly correlated with hepatic LBP expression and plasma LBP levels (*p* < 0.0001, *r* = 0.91; *p* < 0.0001, *r* = 0.93).

### Anorexia is not the main driver of muscle atrophy, disrupted gut-liver homeostasis and microbial dysbiosis in cachectic mice

**Figure 4:**
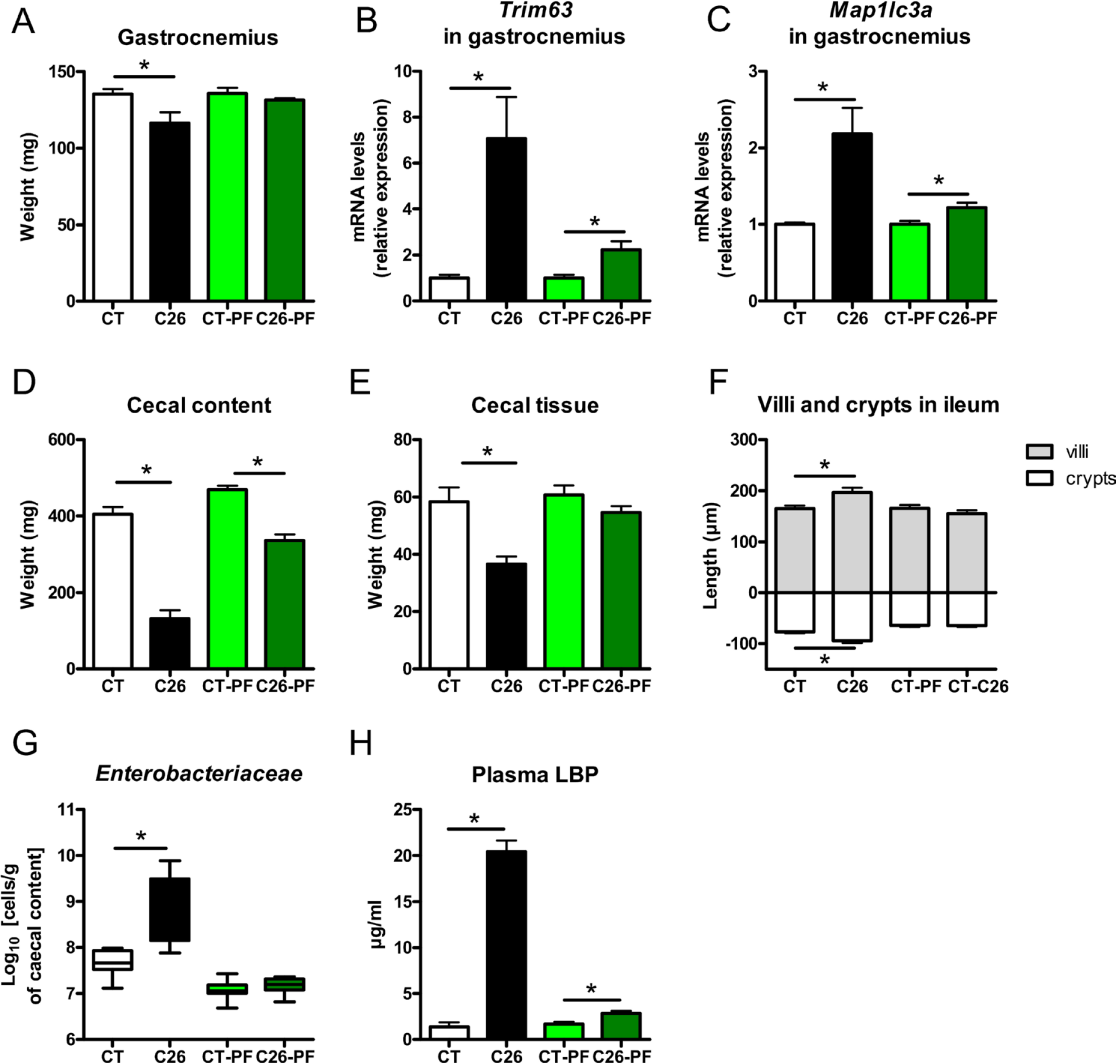
Anorexia is not the main driver of muscle atrophy, intestinal alterations and microbial imbalance in C26 mice (**A**) Gastrocnemius weight. (**B**–**C**) mRNA expression of markers involved in muscle atrophy in the gastrocnemius. (**D**–**E**) Cecal content and tissue weight. (**F**) Villi length and crypt depth in the ileum. (**G**) Cecal *Enterobacteriaceae* levels, as determined by qPCR. (**H**) Plasma LBP levels. Mice were either sham-injected (CT), injected with cancer cells (C26), sham-injected and pair-fed to CT mice (CT-PF) or sham-injected and pair-fed to C26 mice (C26-PF). *n* = 7–8, ^*^p < 0.05. Only four values detected for LBP in the CT group.

We next looked for the mechanisms underlying the microbial dysbiosis and altered gut barrier function. Undernutrition, malnutrition, anorexia and fasting have been shown to be associated with microbial dysbiosis and altered gut barrier function [25–28]. To evaluate the role of the reduced food intake in the intestinal and microbial alterations found in cachectic mice, we repeated the experiment including pair-fed animals. Two groups of healthy mice were pair-fed either to the CT group (CT-PF) or to the C26 group (C26-PF). The CT-PF group was included to control for the stress related to the pair-feeding procedure. Comparing the CT-PF and the C26-PF group allows us to assess strictly the effect of the caloric restriction. Mice pair-fed to cachectic mice (C26-PF) experienced a weight loss similar to the one exhibited by cachectic mice (C26 group, Supplementary Figure 2). However, C26-PF mice lost mainly fat mass whereas cachectic mice lost mainly muscle mass (Figure 4A, Supplementary Figure 2). Muscle atrophy markers were drastically increased in C26 mice and only marginally induced in C26-PF mice (Figure 4B–4C). The pair-feeding experiment also revealed that intestinal alterations such as reduced cecal content and tissue weight, increased villi length and crypt depth and altered expression of markers of gut barrier function could not be attributed to anorexia (Figure 4D–4F, Supplementary Figure 2). Increases in cecal *Enterobacteriaceae*, plasma LBP and hepatic markers of TLR activation were not the consequence of the decreased food intake and anorexia (Figure 4G and 4H, Supplementary Figure 2). Gut microbial changes induced by the pair-feeding were different from the changes observed in cachexia. The only bacterial change found in cachectic mice that could be attributed to reduced food intake was an increase in *Clostridium* cluster XIVa (Supplementary Figure 3). Therefore, we concluded that the reduced food intake alone does not drive the major microbial changes observed in cachectic mice.

### Interleukin-6 drives alterations in the gut-liver homeostasis and microbial changes found in cancer cachexia

**Figure 5:**
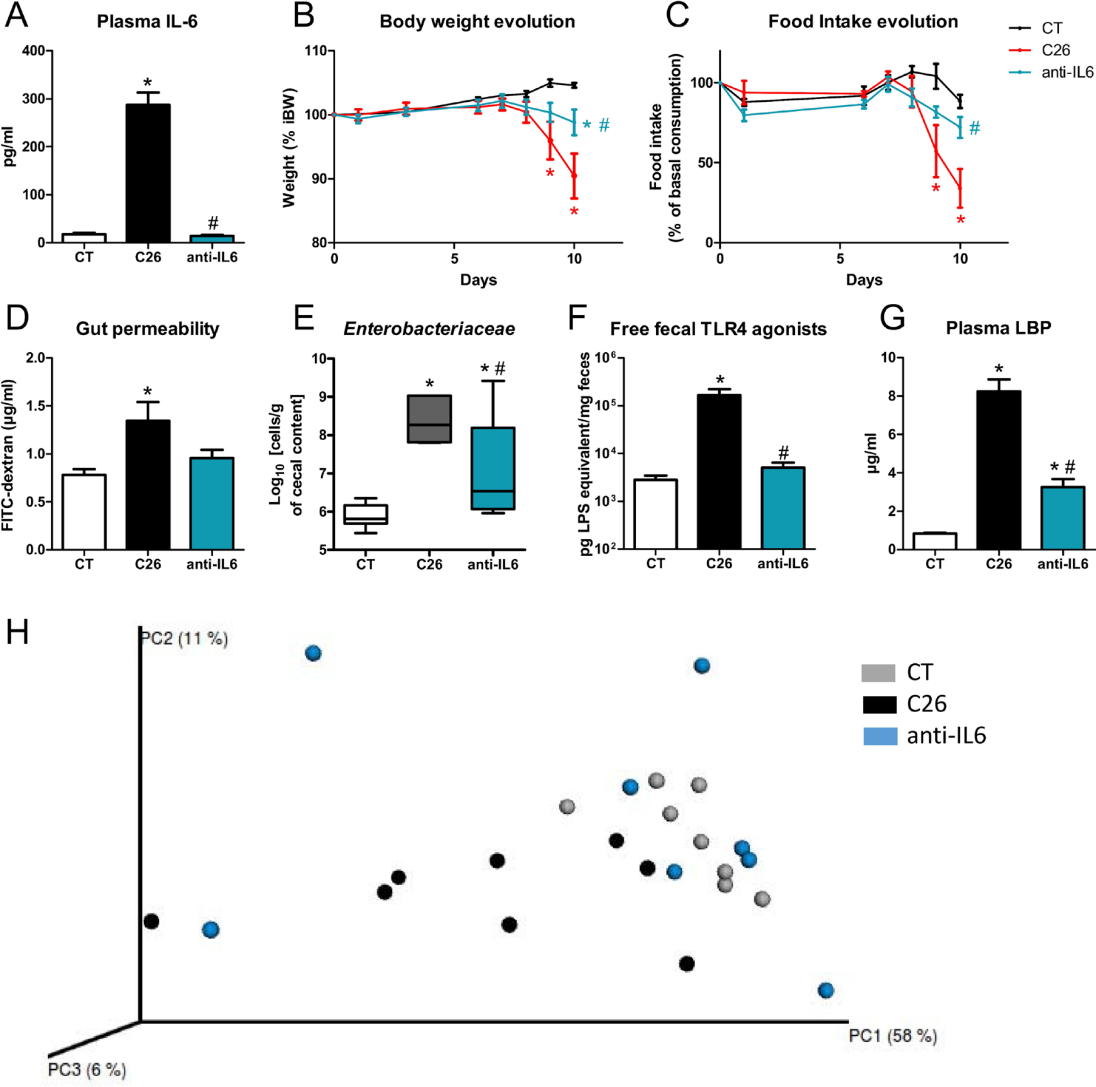
An anti-IL-6 antibody reduces body weight loss, anorexia, gut permeability and microbial alterations (**A**) Plasma IL-6 levels. (**B**–**C**) Body weight and food intake evolution. (**D**) Gut permeability as assessed *in vivo* by FITC-dextran. (**E**) Cecal *Enterobacteriaceae* levels, as determined by qPCR. (**F**) Fecal activity in free TLR4 agonists. (**G**) Plasma LBP levels. (**H**) Principal coordinate analysis of the Morisita-Horn beta-diversity index computed based on the OTU table (adonis permutation test, *R*^2^ = 28.1, *p* = 0.04, meaning the group effect explains 28.1% of the variation in the dataset). Mice received either a sham-injection and a treatment with the vehicle (CT), or an injection of cancer cells and a treatment with the vehicle (C26) or an injection of cancer cells and a treatment with the anti-IL-6 antibody (anti-IL6). *n* = 6–8, ^*^p < 0.05 vs CT, ^#^*p* < 0.05 vs C26.

As the driver for the microbial and intestinal alterations was not the reduced food intake, we looked for a mediator secreted directly by the tumor or by the host in response to the tumor presence. IL-6 has been proposed as a key driver of cachexia in the C26 model, but this remains controversial [29–32]. Interestingly, IL-6 regulates the expression of claudin 2 and intestinal tight junctions [33]. In our hands, plasma IL-6 levels were drastically increased in C26 mice compared to other cytokines (Supplementary Figure 4). Administration of an anti-IL-6 antibody was able to counteract this increased IL-6 level, leading to an almost complete maintenance of body weight and food intake and preventing the induction of markers of muscle atrophy, despite a slight increase in tumor mass (Figure 5A–5C, Supplementary Figure 5). The anti-IL-6 antibody reduced alterations in the gut barrier function, as assessed by FITC-dextran assay and expression of markers of the gut barrier function. The anti-IL-6 antibody also prevented the increase in cecal *Enterobacteriaceae*, free fecal TLR4 agonists and plasma LBP levels as well as the induction of TLR4-related markers in the liver (Figure 5D–5G, Supplementary Figure 5). Such improvements were not observed upon injection of an isotype control (Supplementary Figure 6). Importantly, the anti-IL-6 antibody mitigated the impact that cancer cells have on the gut microbiota composition, as shown in the Principal Coordinate Analysis plot of the Morisita-Horn beta-diversity index (Figure 5H, Supplementary Table 2).

### Gut dysfunction found in cancer cachexia is resistant to teduglutide, a glucagon-like peptide 2 analogue, and *Faecalibacterium prausnitzii,* an anti-inflammatory bacterium with gut barrier-enhancing properties

To investigate the therapeutic interest of an improved gut barrier function in cachexia, we attempted to restore this gut barrier function using teduglutide, a GLP-2 analogue approved for the treatment of short bowel syndrome [34]. As expected, teduglutide treatment increased intestinal cell proliferation as reflected by an increased cecal tissue weight and increased villi length and crypt depth in ileum, with no effect on tumor mass. However, teduglutide treatment did not restore the expression of markers of the gut barrier function (Supplementary Figure 7).

We next sought to investigate the potential of *Faecalibacterium prausnitzii* as an alternative mean to tackle the altered gut homeostasis. *F. prausnitzii* improved gut barrier function in mouse models of colitis [35], chronic low-grade inflammation [36] and partial restraint stress [37]. *F. prausnitzii* is a relevant bacterial target for human intervention as it is a major member of the human microbiota, which is reduced upon chemotherapy in cancer patients (from 9% to below 0.01%) [38]. In cachectic mice, *F. prausnitzii* did not modify the tumor mass, the gut permeability, plasma LBP levels and intestinal markers of the gut barrier function (Supplementary Table 3).

### Serum LBP is an independent predictor of anorexia, cachexia and survival in colorectal and lung cancer patients

**Figure 6:**
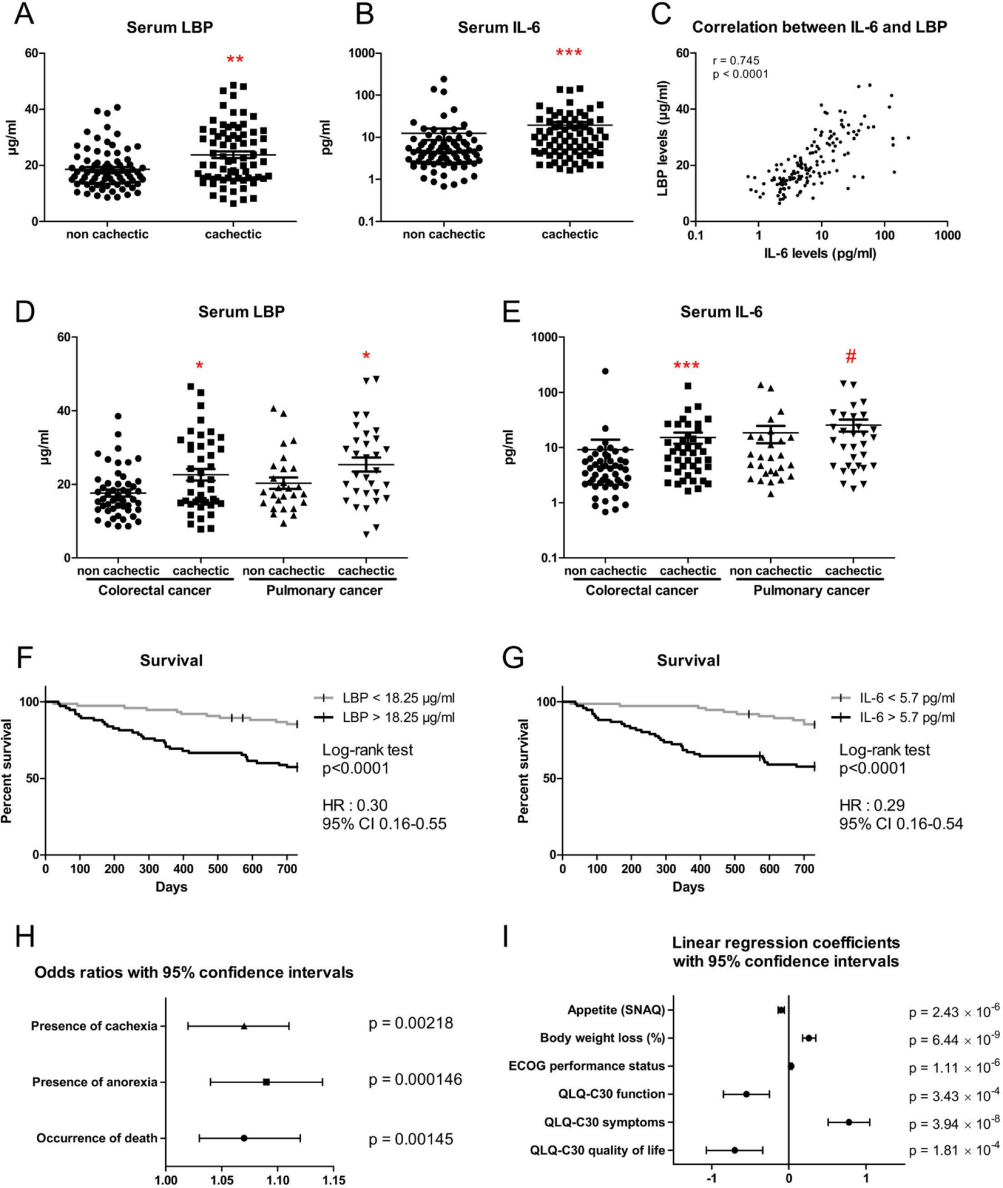
Serum LBP levels are increased in cachectic patients with colorectal cancer and lung cancer (**A**–**B**) Serum LBP and IL-6 levels in cancer patients with or without cachexia (*n* = 78/74 and *n* = 78/73, respectively). (**C**) Correlation between serum LBP levels and serum IL-6 levels in cancer patients with or without cachexia. (**D**–**E**) Serum LBP and IL-6 levels in cancer patients with or without cachexia, stratified according to cancer type (*n* = 51/43/27/31 and *n* = 51/42/27/31, respectively). (**F**–**G**) Kaplan-Meier curve showing the survival fraction in two subpopulations of patients stratified according to their LBP level (*n* = 76/75) or to their IL-6 level (75/75). (**H**) Odds ratios with 95% confidence intervals obtained from regression models with adjustment for sex, age and cancer type. (**I**) Coefficient values with 95% confidence intervals obtained from regression models with adjustment for sex, age and cancer type. Regression coefficients represent the mean change in the response variable for one unit of change in the predictor variable while holding other predictors in the model constant. ^*^p < 0.05, ^**^p < 0.01, ^***^p < 0.001, ^#^*p* = 0.1.

To evaluate the translational value of our findings, we measured serum LBP and IL-6 levels in a cohort of 152 patients suffering from colorectal or lung cancer accompanied or not by cachexia. LBP levels as well as IL-6 levels were higher in cachectic patients than in non-cachectic patients, with both parameters showing a strong correlation (Figure 6A–6C). Similar results were obtained when patients were stratified by cancer type (Figure 6D and 6E). Patients were also stratified in a low-LBP population and a high-LBP population based on the median LBP value. Patients within the high-LBP population had a 3-time lower survival rate than patients within the low-LBP population (Figure 6F). Similar results were observed when stratifying the patients according to their IL-6 levels (Figure 6G). Last but not least, multivariate analyses revealed that the LBP level is a powerful predictive factor for death occurrence, cachexia and anorexia presence, appetite, body weight loss, functional status, symptoms and quality of life when data were adjusted for age, sex and cancer type (Figure 6H and 6I). For instance, such analyses predict that for each one-unit increase in LBP level, the odds of being dead, of experiencing anorexia and of suffering from cachexia increase by 7%, 9% and 7%, respectively. Similar results were obtained after adjustment for age, sex, cancer type and cancer stage (Supplementary Table 4).

## DISCUSSION

Chemotherapy has long been considered the main driver of the disruption of the gut barrier function and gut microbiota composition observed in cancer patients. Here, we show that the gut barrier function and the gut microbiota composition and function are altered in a mouse model of cancer cachexia independently of any chemotherapy. The gut barrier function of cachectic mice was impaired at various levels: altered intestinal morphology, decreased renewal for various cell linages, depressed immune system, and increased gut permeability associated with decreased expression of tight junctions. These multiple injuries to the gut barrier sign the existence of a complex yet coordinated interplay between the gut barrier function and cancer cells, independently of any anticancer treatment. Of note, signs of a reduced gut barrier function were also found in leukemic mice with cachexia [18] and in a mouse model of colon cancer cachexia [39]. Furthermore, a study of 16 acute myeloid patients also reported an increased gut permeability before any chemotherapy [40], raising the likelihood that our findings may be relevant to human pathology. Altogether, our data point out cancer presence as one previously unsuspected partner driving gut dysfunction.

The gut microbiota was deeply altered in cachectic mice at the taxonomical and functional levels. The *Enterobacteriaceae* level, as well as the fecal content in free TLR4 agonists, were consistently increased in cachectic mice. Importantly, these alterations were not due to the reduced food intake and were partially counteracted by the anti-IL-6 treatment, suggesting that, as for the gut barrier function, cancer cells and associated increased IL-6 levels contribute to the gut microbial dysbiosis. This set of experiments clearly shows the host's prominent role in shaping the gut microbiota in cancer cachexia.

*Enterobacteriaceae* family members are Gram-negative bacteria presenting LPS at their surface. LPS, among other microbial molecules, can lead to the activation of the TLR4 receptor. LBP will bind the lipid A moiety of LPS and depending of LBP level, will enhance or inhibit the cell response to LPS through the CD14-MD2-TLR4 complex [22]. While LBP is named after its ability to bind to LPS, it can also recognize other bacterial compounds, such as lipopeptide. In our hands, LBP levels were increased in two populations of cancer cachectic patients versus cancer non-cachectic patients. The level of this acute-phase protein was predictive of death occurrence and cachexia presence, and a significant contributor in the modeling of several cachectic features. In accordance with our findings, another study found out that LBP levels tended to be increased in lung cancer patients losing weight compared to weight-stable lung cancer patients [41]. The lack of statistical significance may ensue from the low number of subjects enrolled in the study (*n* = 10 per group). The role of LBP in inflammatory disorders is complex and unclear. Limited progress has been achieved lately in understanding the function of LBP, likely due to a research focus toward the TLR system [22]. Currently, based on our data, we can propose LBP to be considered a predictive biomarker for cancer cachexia.

LBP is mainly secreted by the liver. Hepatic *Lbp* expression is controlled both by the gut microbiota [42] and IL-6 [43], making it a unique interface between inflammation and gut microbiota. Administration of an anti-IL-6 antibody decreased IL-6 levels to levels found in control mice and prevented 67% and 60% of the cancer-induced increase in plasma LBP levels and hepatic *Lbp* expression, respectively, meaning that IL-6 levels are responsible, at least partially, for the increased plasma LBP levels and hepatic *Lbp* expression. This observation in mice is further supported by our findings in humans, showing a strong correlation between serum IL-6 and LBP levels.

Previous studies showed that IL-6 or IL-6 receptor antibodies can counteract, at least in part, body weight loss, fat browning and/or muscle atrophy in mouse models of cachexia [29, 44–46] with contradicting effects on tumor progression [45, 47]. Here, we confirmed that the anti-IL-6 antibody reduces body weight loss and muscle atrophy. In addition, we discovered that IL-6 links muscle atrophy with gut barrier dysfunction and microbial dysbiosis. So far, it remains unclear if the improvement of the gut barrier function by the anti-IL6 antibody is a direct consequence of the neutralization of IL-6 or a consequence of an improvement of the cachectic state. The work of Suzuki *et al*., among others, is in favour of a direct link between IL-6 and the gut barrier function. These authors showed that IL-6 increases tight junction permeability by stimulating the expression of claudin 2 [33]. In accordance with this report, in our studies, claudin 2 expression was increased in the jejunum of cachectic mice and this increase was blocked by the anti-IL-6 treatment. To further clarify this question, it would be interesting to evaluate the impact on the gut barrier function of anticachectic agents with no impact on IL-6 levels.

If the baseline increase in gut permeability, independently of any drug treatment, plays a role in controlling cancer progression and cachexia development remains to be determined. Gut barrier alterations may lead to a translocation of pro-inflammatory bacterial compounds, thereby reinforcing systemic inflammation, a main driver of several cachectic features. In this case, restoring gut barrier function may bring about benefits. One lesson from our study is that gut dysfunction found in cancer cachexia is resistant to teduglutide and *F. prausnitzii*, two therapeutic tools previously used to improve gut barrier function. Therefore, new therapeutic tools targeting the gut barrier function are warranted and will need to be tested in the context of cancer cachexia. Modulating the gut microbiota composition in order to improve gut function could be an interesting therapeutic avenue. Previous work from our lab suggest that administration of microbiota-modulating dietary ingredients improves intestinal homeostasis and confers benefits in a mouse model of leukemia and cachexia [18, 19]. Based on our current work, pharmacological tools, such as antibodies targeting IL-6, may also represent a successful approach to tackle microbial changes and gut barrier dysfunction. In a clinical setting, such modulation of the gut barrier function and the microbial ecosystem will need to be designed taking into account our continuously increasing knowledge of the contribution of the gut microbiota to chemotherapy efficacy and toxicity [3, 48]. Currently, we can say that the impact of a modulation of the gut barrier function on drug efficacy likely differs from one drug to another. For instance, gut barrier alterations induced by cyclophosphamide is essential for the efficacy of the drug [49]. This does not seem to be the case for anti-CTLA4 cancer therapy, as feeding *Bacteroides fragilis* and *Burkholderia cepacia* to microbiota-depleted mice decreases the extent of intestinal damage and colitis while restoring the therapeutic response to anti-CTLA4 [50].

In conclusion, our data clearly establish that the gut barrier function, as well as the gut microbiota composition and function, are consistently altered in cancer cachexia independently of any chemotherapy. We discovered that the serum level of LBP, often presented as a reflection of the bacterial antigen load, was increased in cachectic mice and we confirmed this finding in patients. In addition, we found out that serum LBP was predictive of cachexia and death. Importantly, we demonstrated that these alterations in gut microbiota and gut barrier function are related to the increased level of IL-6 rather than to the anorexia. Classical gut barrier function enhancers, such as the GLP-2 analogue teduglutide and *F. prausnitzii*, were unsuccessful to restore this gut barrier function, whereas IL-6 antibody improves the gut barrier function and the microbial dysbiosis, as well as muscle atrophy, anorexia and body weight loss. We believe these findings are not only of mechanistic significance, but also of great importance for the therapeutic management of cancer cachexia, pointing out to the gut and its inhabitants as new key partners in cancer cachexia.

## MATERIALS AND METHODS

### Cell and bacterial cultures

Colon carcinoma 26 (C26) cells and *Faecalibacterium prausnitzii* A2–165 (DSM 17677) were grown as described [18, 36]. Bacterial culture was centrifuged, supernatant was removed, pelleted bacteria were suspended in culture medium with 15% glycerol and aliquots of 700 μl were stored at −80° C. Bacterial numeration was performed after thawing by plating on supplemented YBHI agar in an anaerobic chamber.

### Animals

Male CD2F1 mice (7 weeks old, Charles River Laboratories, Italy) were housed in individually ventilated or filter-top cages with a 12 h light/dark cycle and fed an irradiated chow diet (AO4-10, 2.9 kcal/g, Safe, France). After one week acclimatization, either a saline solution or C26 cells (1 × 10^6^ cells in 0.1 ml saline) were injected subcutaneously. All C26-injected mice displayed a tumor mass observable since day 7. Food intake and body weight were recorded. Eight mice were randomly assigned in each group based on their body weight on the day of cell injection.

The pair-feeding experiment was composed of 4 groups of mice: CT group (sham-injected and fed *ad libitum*), C26 group (receiving an injection of C26 cancer cells and fed *ad libitum*), CT-PF group (sham-injected and fed the mean amount consumed by the CT mice) and C26-PF group (sham-injected and fed the mean amount consumed by the C26 mice). Pair-fed mice received daily in 2 equal portions the amount of food consumed by the group they were matched to, with one week delay (Supplementary Figure 8).

300 μg monoclonal rat anti-murine IL-6 antibody (clone MP5-20F3, BioXCell, NH, USA), 300 μg rat IgG1 isotype control (catalogue # BE0088, BioXCell, NH, USA) or vehicle (phosphate-buffered solution) was injected subcutaneously on days 7 and 9. Human [gly2] glucagon-like peptide 2 (GLP-2), also known as teduglutide (Pepceuticals Inc, England), was dissolved in sterile degassed PBS and 2.5 μg of the compound, or PBS, was subcutaneously injected twice a day [51]. *F. prausnitzii* or vehicle was administered daily by oral gavage (10^9^ CFU in 200 μl) from day 1 to 9.

Ten days after cancer cell injection, fresh feces were collected, mice were fasted from 7AM to 1PM (except for one experiment) and portal and systemic blood as well as tissue samples and cecal content were harvested following anaesthesia (isoflurane gas, Abbot, Belgium). Blood was centrifuged. Tissues were weighed and frozen in liquid nitrogen, with intestinal sections stored in 4% formaldehyde. All of the samples were stored at −80° C.

Fluorescein Isothiocyanate (FITC)-Dextran 4 (Sigma-Aldrich, MO, USA; 600 mg/kg, 125 mg/ml PBS) was administrated by oral gavage one hour before blood sampling. Serum fluorescence was assessed using a Victor-X2 plate reader (Perkin Elmer, MA, USA) at 485/535 nm. FITC-dextran was diluted in naïve serum plasma premixed with PBS (1:1 v/v) to generate a standard curve.

The experiments were approved by and performed in accordance with the guidelines of the local ethics committee. Housing conditions were as specified by the Belgian Law of 29 May 2013, regarding the protection of laboratory animals (agreement no LA1230314).

### Tissue mRNA analyses

The isolation of RNA, preparation of complementary cDNA and real-time polymerase chain reaction were performed as previously described [18] (primer sequences in Supplementary Table 1).

### Biochemical and histological analyses

Plasma cytokines were measured using a customized multiplex kit (Bio-Rad, Nazareth, Belgium) and Luminex technology (Bio-Plex, Bio-Rad). Human and mouse LBP levels, as well as human IL-6 levels, were assessed using ELISA kits (HycultBiotech, PA, USA, and R&D Systems, Oxon, UK). One patient was excluded from the IL-6-related analyses due to an out-of-range value. Alkaline phosphatase activity was determined using a method adapted from Bessey and colleagues [52]. Haematoxylin-eosin stained sections were digitalized at a 20× magnification using a SCN400 slide scanner (Leica, Wetzlar, Germany). Crypt depth and villi length were manually measured by an investigator blinded for treatments using the Leica Image Viewer Software (Version 4.0.7; at least 10 measures of each parameter per section and two independent sections per mouse).

### Detection of free fecal TLR4 agonists

Fecal TLR4 agonists were measured using a HEK-Blue reporter cell line according to manufacturer instructions (InvivoGen, France). Fecal material was suspended in LAL water (Lonza, MD, USA) to a concentration of 100 mg/ml and homogenized for 4 min using a Tissue Lyzer without beads. Samples were centrifuged and supernatant was serially diluted, heated at 56° C for 45 min and applied to cells. Escherichia coli LPS (Sigma-Aldrich) was used to generate a standard curve. After 21 h of stimulation, cell culture supernatant was mixed to QUANTI-Blue medium (Invivogen) for 3 h and absorbance was measured at 620 nm. A control cell line (HEK-Blue Null1 cells) was included to remove unspecific signals.

### Gut microbiota analyses

Genomic DNA was extracted from the cecal content using a QIAamp DNA Stool Mini Kit (Qiagen, Germany), including a bead-beating step. Absolute quantification of the *Enterobacteriaceae* family was performed using qPCR (primers presented in Supplementary Table 1). The samples were PCR-enriched for the V5–V6 region of the 16S rRNA gene and then underwent a library tailing PCR (primers in Supplementary Table 1). The amplicons were purified, quantified and sequenced using an Illumina MiSeq to produce 2 × 300 bp sequencing products. Initial quality-filtering of the reads was conducted with the Illumina Software, yielding an average of 110 006 pass-filter reads per sample. Quality scores were visualized, and reads were trimmed to 220 bp (R1) and 200 bp (R2). The reads were merged with the merge-Illumina-pairs application [53]. For samples with >25000 merged reads (all samples but four), a subset of 25000 reads was randomly selected using Mothur 1.32.1 [54]. The UPARSE pipeline implemented in USEARCH v7.0.1001 [55] was used to further process the sequences. Putative chimaeras were identified against the Gold reference database and removed. Clustering was performed with a 98% similarity cut-off to designate operational taxonomic units (OTUs). Non-chimeric sequences were also subjected to taxonomic classification using the RDP MultiClassifier 1.1 from the Ribosomal Database Project [56]. The phylotypes were computed as percent proportions based on the total number of sequences in each sample. Beta-diversity indexes and Adonis values were calculated using QIIME [57]. PCoA plot of the beta-diversity indexes were obtained using EMPeror [58]. The LDA effect size was computed and plotted using LEfSe [59]. Sequences can be found in the MG-RAST database (projects ID Cachexia_1, Cachexia_pair_feeding, Cachexia_IL6).

### Cross-sectional prospective study with cancer patients

The cohort of patients and its characterization was previously reported [60]. This cross-sectional prospective study was performed at the Cliniques universitaires Saint-Luc, Brussels, Belgium. The protocol was approved by the ethics committee of the Université catholique de Louvain (NCT01604642). Patients with colorectal or lung cancer, confirmed by anatomopathology, were recruited at the diagnosis or at relapse, before any therapeutic intervention, from January 2012 to March 2014. Written consent was given prior to entry into the study. Exclusion criteria were: non-caucasian subjects, obvious malabsorption, major depression, artificial nutrition, high doses of steroids (>1 mg/kg hydrocortisone equivalent), hyperthyroidism, other causes of malnutrition, major walking handicap, ECOG performance status ≥4 and psychological, familial, social or geographic conditions that would preclude participation in the full protocol. The cachectic status was determined according to the definition proposed by Fearon *et al*, as an involuntary weight loss > 5% over the past 6 months or weight loss > 2% and body mass index < 20 kg/m^2^ or weight loss > 2% and low muscularity (LM) [61]. Overall survival was analyzed since the day of the inclusion visit to 24 months later. Anorexia was evaluated by the Simplified Nutritional Appetite Questionnaire (SNAQ) score and was defined by a SNAQ score < 14 [62]. The functional status was assessed by two previously validated scales, namely Eastern Cooperative Oncology Group (ECOG) and EORTC QoL questionnaire (QLQ-C30) [63].

### Statistical analyses

Outliers were removed using the Grub's test. The statistical significance of differences between groups was assessed using Student's *t*-test when comparing two groups (Mann-Whitney test for human data), or one-way ANOVA followed by Tukey's multiple comparison tests when comparing several groups. Two-way ANOVA followed by Bonferroni post-tests was used to assess the significance of two independent variables for one dependent variable. The data are presented as bar graphs with standard error of the mean, whiskers plots with maximum and minimum or scatter plots. Statistical analyses were performed using GraphPad Prism 5.0 and R [64]. *P* < 0.05 was considered statistically significant.

## SUPPLEMENTARY MATERIALS FIGURES AND TABLES





## Abbreviations

C26: colon carcinoma 26
Cd: cluster of differentiation
Clnd2: claudin 2
CT: control
Ctsl: cathepsin L
ELISA: enzyme-linked immunosorbent assay
Fbxo32: F-box protein 32, alias atrogin 1
FITC: fluorescein isothiocyanate
Foxp3: forkhead box p3
GLP-2: glucagon-like peptide 2
Ido1: indoleamine 2,3-dioxygenase
IL: interleukin
Itgax: integrin alpha X, alias CD11c
Klf4: Kruppel-like factor 4
LBP: lipopolysaccharide-binding protein
Lgr5: leucine-rich repeat-containing G-protein coupled receptor 5
LPS: lipopolysaccharide
Map1lc3a: microtubule associated protein 1 light chain 3 alpha, alias LC3
Ocln: occludin
PF: pair-fed
qPCR: quantitative polymerase chain reaction
Tcf4: transcription factor 4
Ted: teduglutide
Tjp1: tight-junction protein 1, alias zonula occludens 1
TLR: toll-like receptor
Tnf: tumor necrosis factor alpha
Trim63: tripartite motif-containing 63, alias MuRF1
