# Supplementary material for: Increased gut permeability in cancer cachexia: mechanisms and clinical relevance
Source: Oncotarget. 2018 Apr 6;9(26):18224–38. doi: 10.18632/oncotarget.24804 (PMC5915068; doi:10.18632/oncotarget.24804)
Supplement: Supplementary file 1 [file oncotarget-09-18224-s001.pdf]

Increased gut permeability in cancer cachexia: mechanisms and clinical relevance

SUPPLEMENTARY MATERIALS

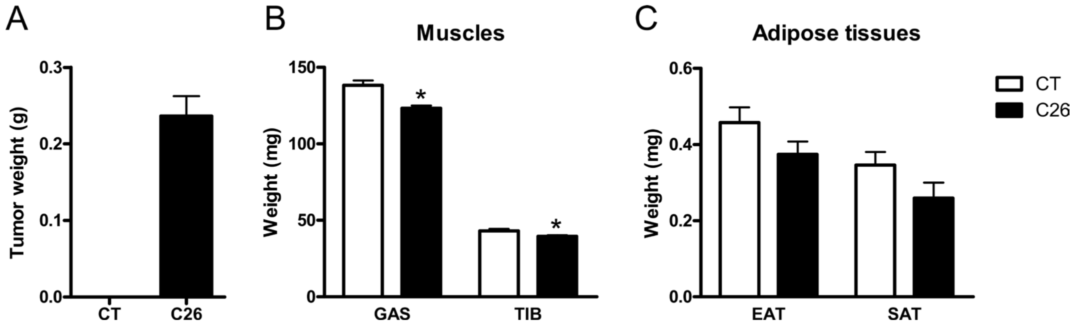

Supplementary Figure 1: Muscle atrophy and fat mass loss in a mouse model of cancer cachexia. (**A**) Tumor mass. (**B**) Gastrocnemius (GAS) and tibialis (TIB) muscle weight. (**C**) Epididymal (EAT) and subcutaneous (SAT) adipose tissue weight. CT: sham-injected mice; C26: cachectic mice. $n = 8$, $^*p < 0.05$.

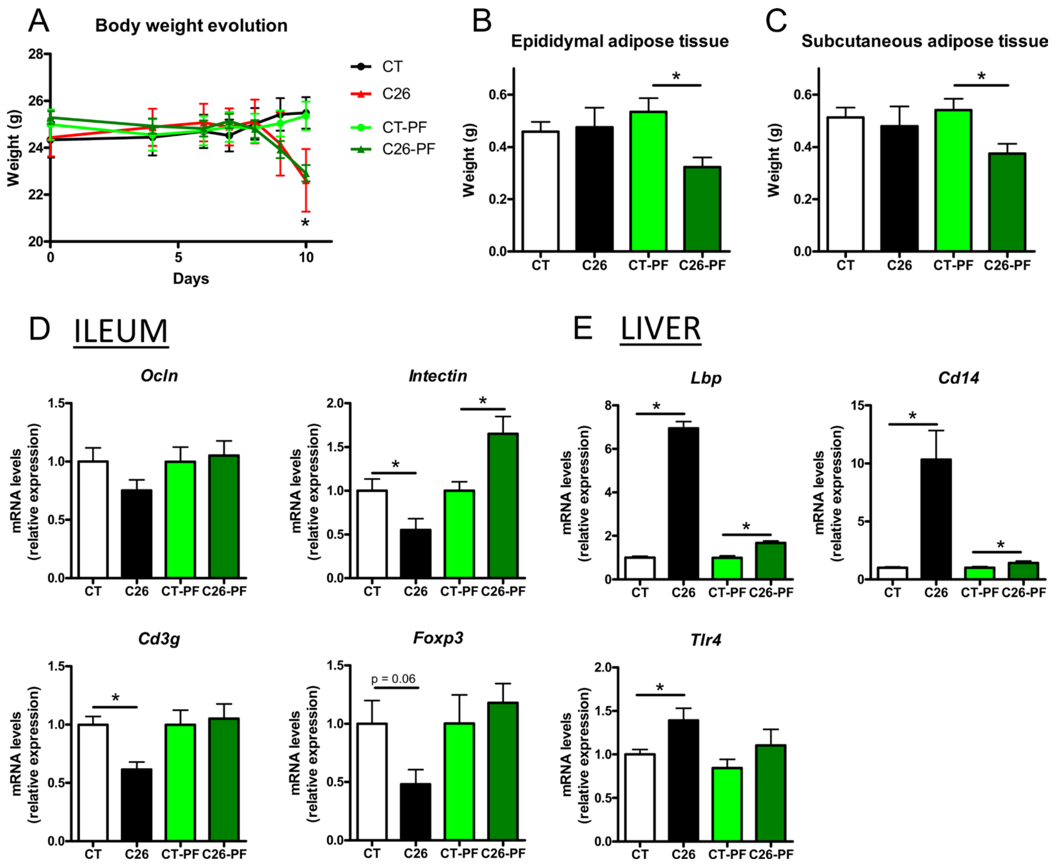

Supplementary Figure 2: Anorexia is not the main driver of the metabolic and intestinal alterations in cachectic mice. (**A**) Body weight evolution in sham-injected mice (CT), cachectic mice (C26), sham-injected mice pair-fed to CT mice (CT-PF) and sham-injected mice pair-fed to C26 mice (C26-PF). (**B–C**) Epididymal and subcutaneous adipose tissue weights. (**D**) mRNA expression in the ileum of markers involved in gut barrier function. (**E**) mRNA expression in the liver of markers related to the TLR4 pathway. $n = 6–8$, $^*p < 0.05$. Tumor mass for this experiment was 340 ± 34 mg ($n = 7$, tumor mass in the C26 group only).

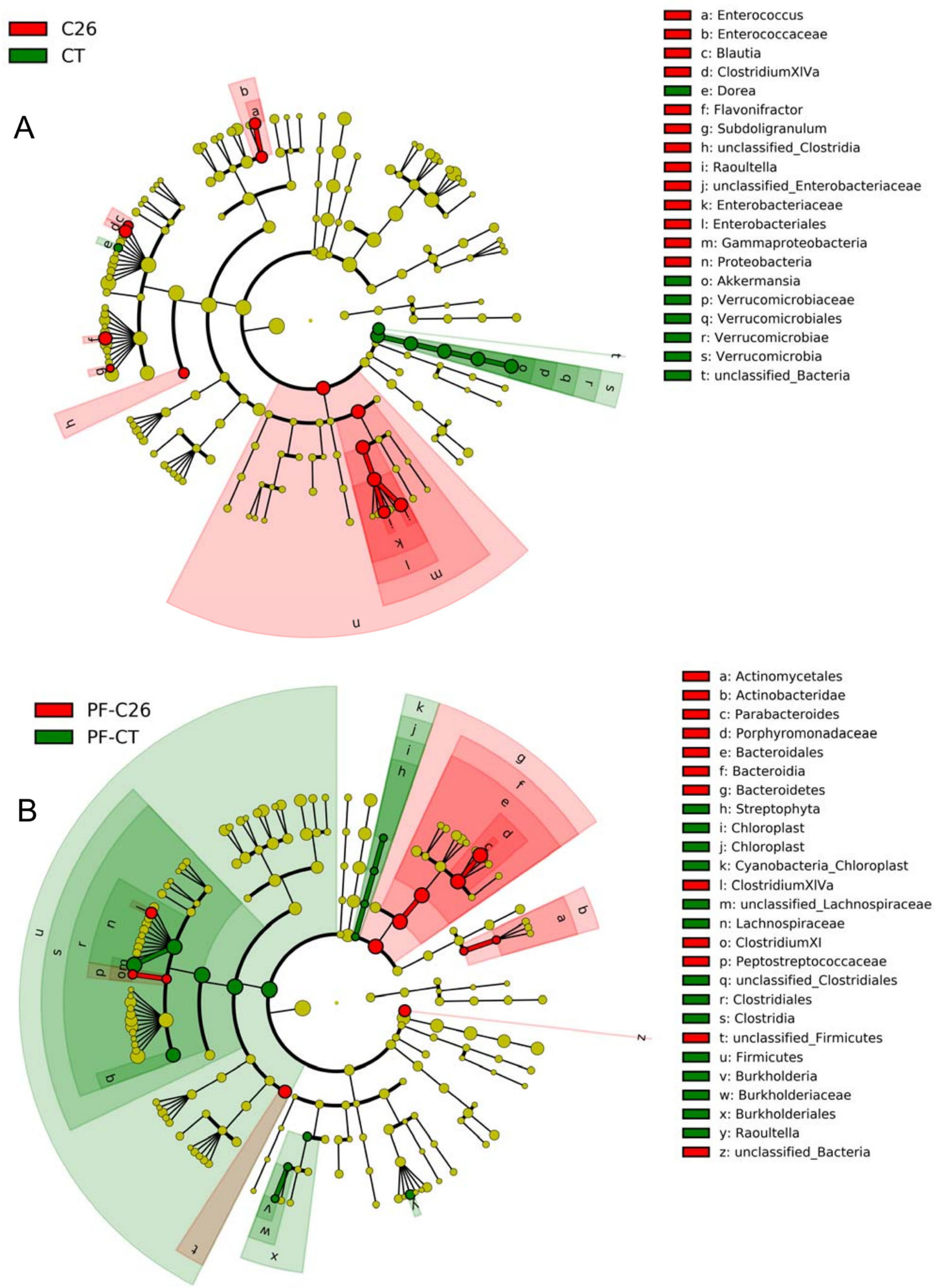

Supplementary Figure 3: Anorexia is not the main driver of gut microbial changes in cachectic mice. (**A**) LEfSe cladogram in red for the taxa enriched in cachectic mice (C26) and in green for the taxa enriched in sham-injected mice (CT). (**B**) LEfSe cladogram in red for the taxa enriched in mice pair-fed to the cachectic group (C26-PF) and in green for the taxa enriched in mice pair-fed to the sham-injected group (CT-PF). *n* = 7–8.

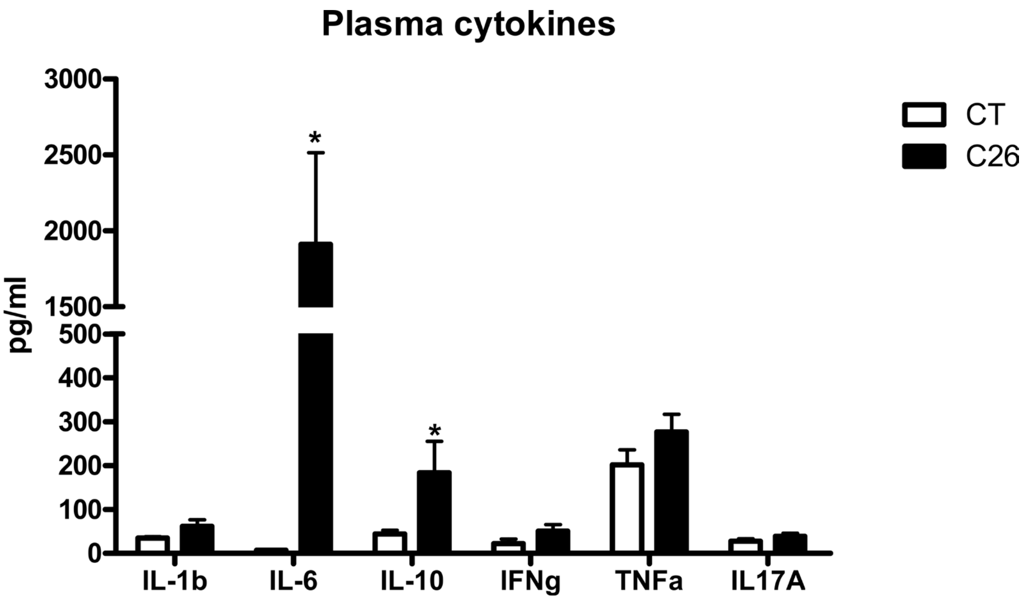

Supplementary Figure 4: IL-6 is increased in cachectic mice. Plasma cytokine levels in sham-injected mice (CT) and cachectic mice (C26) 11 days after injection. $^{*}p < 0.05$, t-test performed on log-transformed data.

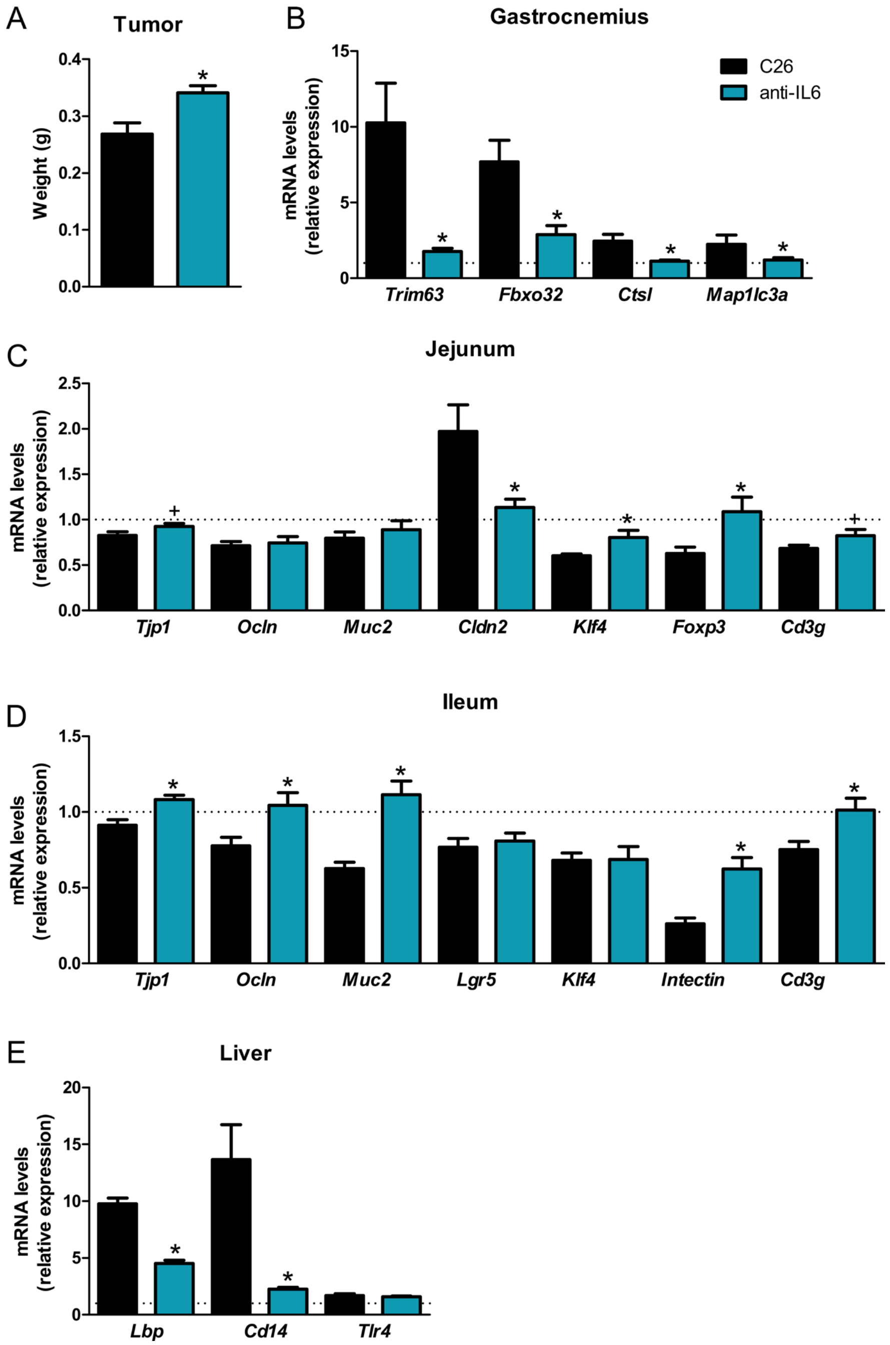

Supplementary Figure 5: Administration of an anti-IL-6 antibody blunted muscle atrophy, partially restored the expression of markers involved in gut barrier function, and prevented the induction of TLR4-related markers in the liver. (**A**) Tumor weight. mRNA expression of markers related to muscle atrophy (**B**), gut barrier function (**C**, **D**) and the TLR4 pathway (E) in cachectic mice treated with the vehicle (C26, black) and cachectic mice treated with the anti-IL-6 antibody (anti-IL6, blue). $n = 6–8$, $^{+}p < 0.1$, $^{*}p < 0.05$. The dotted line indicates the expression level in sham-injected mice.

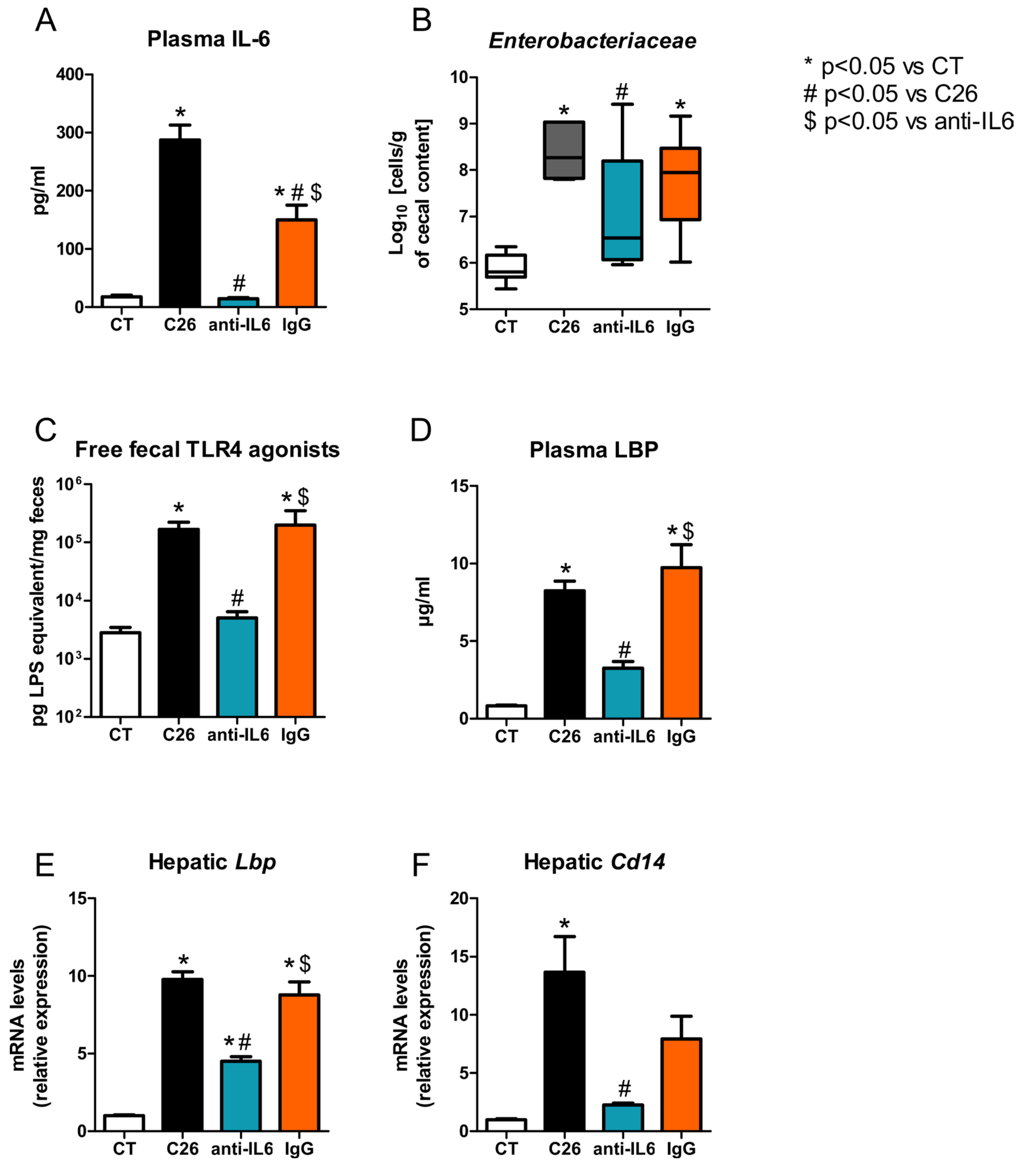

Supplementary Figure 6: Administration of an anti-IL-6 antibody blunted the increase in Enterobacteriaceae levels, free fecal TLR4 agonists, plasma LBP and hepatic Lbp and Cd14 expression, with no effect of the isotype control. (**A**) Plasma IL-6 levels, (**B**) cecal *Enterobacteriaceae* levels, (**C**) free fecal TLR4 agonists, (**D**) plasma LBP levels and (**E**–**F**) hepatic mRNA expression of markers related to the TLR4 pathway (D) in sham-injected mice (CT), cachectic mice treated with the vehicle (C26), cachectic mice treated with the anti-IL-6 antibody (anti-IL6) and cachectic mice treated with an isotype control (IgG). n = 6–8.

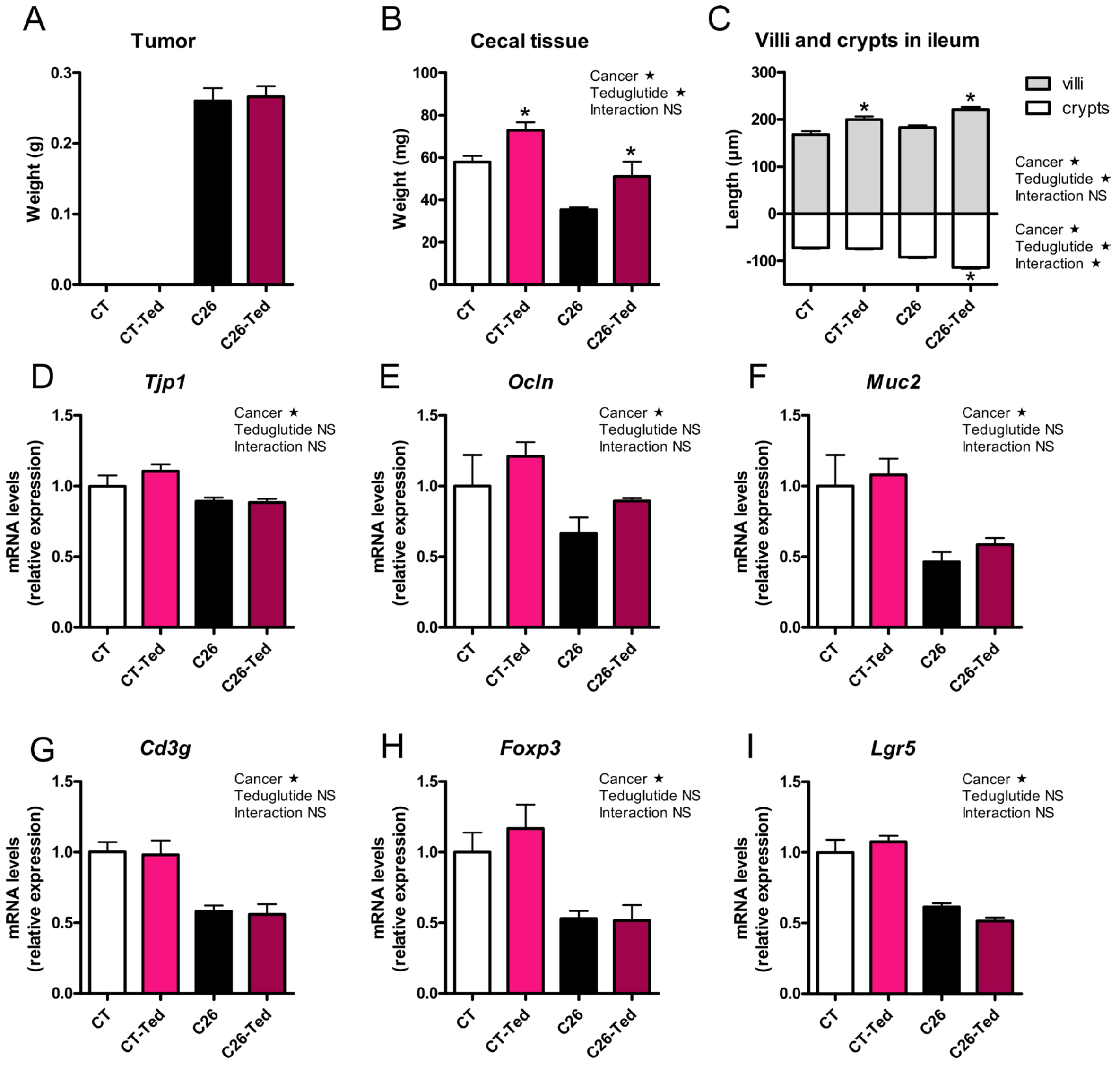

Supplementary Figure 7: Teduglutide administration did not restore the gut barrier function. (**A**–**B**) Tumor mass and cecal tissue weight. (**C**) Villi length and crypt depth in ileum. (**D**–**I**) mRNA expression of markers involved in gut barrier integrity, gut immunity and cell renewal in the ileum. Mice received either one sham-injection followed by injections of saline solution twice a day (CT), one sham-injection followed by injections of teduglutide twice a day (CT-Ted), one injection of cancer cells followed by injections of saline solution twice a day (C26) or one injection of cancer cells followed by injections of teduglutide twice a day (C26-Ted). *n* = 7–8, $^{*}p < 0.05$ vs saline counterparts, NS not significant.

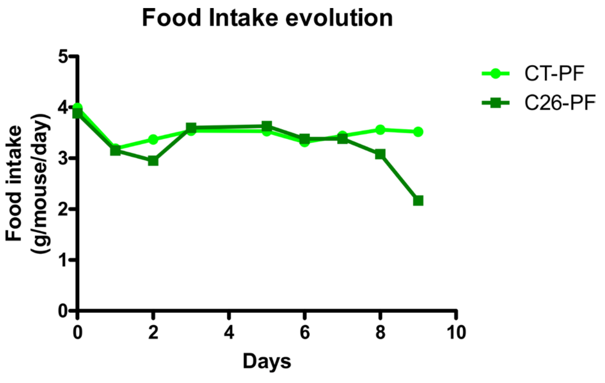

Supplementary Figure 8: Amount of food fed to pair-fed mice. Food intake evolution in sham-injected mice pair-fed to CT mice (CT-PF) and sham-injected mice pair-fed to C26 mice (C26-PF).

Supplementary Table 1: Primer sequences used for RT-qPCR, qPCR, and sequencing assays

Target name	Alias/full name	Forward	Reverse
RPL19 (housekeeping gene)		GAAGGTCAAAGGGAATGTGTTCA	CCTTGTCTGCCTTCAGCTTGT
Fbxo32	atrogin 1, MAFbx, F-box protein 32	ATGCACACTGGTGCAGAGAG	TGTAAGCACACAGGCAGGTC
Ctsl	cathepsin L	GTGGACTGTTCTCACGCTCAAG	TCCGTCCTTCGCTTCATAGG
Itgax	integrin alpha X, CD11c	ACGTCAGTACAAGGAGATGTTGGA	ATCCTATTGCAGAATGCTTCTTTACC
Cd14	CD14	CCTGCCCTCTCCACCTTAGAC	TCAGTCCTCTCTCGCCCAAT
Cd3g	CD3g	TCTCTACTGGGCTCTCTCCAA	CCATCTCCAAGGAAACCAAC
Cldn2	claudin 2	AAGGTGCTGCTGAGGGTAGA	AGTGGCAGAGATGGGATTTG
Foxp3	forkhead box P3	TCCTTCCCAGAGTTCTTCCA	CGAACATGCGAGTAAACCAA
Ido1	indoleamine 2,3-dioxygenase 1	ACCCAGACACGTTTTTCCAC	GCCAGCCTCGTGTTTTATTC
Il15	interleukin 15	TGCAATGAACTGCTTTCTCCT	TCCAGCTCCTCACATTCCTT
Intectin	intectin	GTTGCCCCTGATTCTGCTGG	GCACTATTGCAGAGGTCCGT
Mki67	Ki67	CAGACTTGCTCTGGCCTACC	GGTTGGCGTTTCTCCTCTTT
Klf4	Kruppel-like factor 4	AGAGGAGCCCAAGCCAAAGAGG	CCACAGCCGTCCCAGTCACAGT
Lbp	LBP	GTCCTGGGAATCTGTCCTTG	CCGGTAACCTTGCTGTTGTT
Map1lc3a	LC3	CACTGCTCTGTCTTGTGTAGGTTG	TCGTTGTGCCTTTATTAGTGCATC
Lgr5	Gpr49	TTGGAGAAAGGAGAGCTGGA	AGTGGGACGATCACGAGAAG
Muc2	mucin 2	ATGCCCACCTCCTCAAAGAC	GTAGTTTCCGTTGGAACAGTGAA
Trim63	MuRF1	ACGAGAAGAAGAGCGAGC	CTTGGCACTTGAGAGGAA
Ocln	occludin	ATGTCCGGCCGATGCTCTC	TTTGGCTGCTCTTGGGTCTGTAT
Tcf4	transcription factor 4	ATGGCAAACAGAGGAACTGG	GCCTGCTGAGAGTGAAGGAG
Tlr4	TLR4	CCCTCAGCACTCTTGATTGC	TGCTTCTGTTCCTTGACCCA
Tnf	TNF-alpha	AGCCCCCAGTCTGTATCCTT	GGTCACTGTCCCAGCATCTT
Tjp1	ZO1	TTTTTGACAGGGGGAGTGG	TGCTGCAGAGGTCAAAGTTCAAG
Enterobacteriaceae*		CATTGACGTTACCCGCAGAAGAAGC	CTCTACGAGACTCAAGCTTGC

*as reported by Bartosh et al, Appl Env Microb 2004.

Primers for 16S sequencing	
V5F_Nextera	TCGTCGGCAGCGTCAGATGTGTATAAGAGACAGRGGATTAGATACCC
V6R_Nextera	GTCTCGTGGGCTCGGAGATGTGTATAAGAGACAGCGACRRCCATGCANCACCT
Forward indexing primer	AATGATACGGCGACCACCGAGATCTACAC[i5]TCGTCGGCAGCGTC
Reverse indexing primer	CAAGCAGAAGACGGCATACGAGAT[i7]GTCTCGTGGGCTCGG

16S-specific portion is indicated in bold, and the p5 and p7 flow cell adapters are shown in italics.

Supplementary Table 2: Taxa and OTUs significantly affected by C26 cancer cell presence and/or the injection of antibody targeting IL-6. See supplementary Table 2

Supplementary Table 3: Administration of faecalibacterium prausnitzii did not restore the gut barrier function

	CT			C26				C26-*F. prausnitzii*			
Gut permeability, as assessed by FITC-dextran (µg/ml)	0.63	±	0.10	1.21	±	0.16	*	1.02	±	0.18	
Plasma LBP levels (µg/ml)	1.30	±	0.01	21	±	1.86	*	16.4	±	1.59	*
Tumor mass (mg)	N.A.			233	±	14		216	±	14	
mRNA expression in ileum as assessed by qPCR											
Ocln	1.00	±	0.12	0.69	±	0.07		0.67	±	0.05	
Tjp1	1.00	±	0.06	0.83	±	0.03	*	0.89	±	0.04	
Muc2	1.00	±	0.15	0.75	±	0.06		0.86	±	0.12	
Cd3g	1.00	±	0.13	0.82	±	0.09		0.70	±	0.06	
Foxp3	1.00	±	0.15	0.65	±	0.09		0.58	±	0.08	*
Intectin	1.00	±	0.14	0.27	±	0.06	*	0.42	±	0.10	*

Tumor mass and gut barrier assessment in cachectic mice receiving *Faecalibacterium prausnitzii* or the vehicle by daily gavage. N.A. not applicable. Data are presented as mean ± SEM, *$p<0.05$ vs CT, n=6-8, except for plasma LBP levels (7/5/7 with 5 values not detected in the CT group).

Supplementary Table 4: The LBP level is a predictive factor for death, anorexia and cachexia

**(A) LBP predictive value adjusted for age, sex, cancer type**						
Outcome	regression model	OR	95% CI		p-value	
occurrence of death	logistic	1.07	1.03	1.12	0.00218	**
presence of anorexia	logistic	1.09	1.04	1.14	0.000146	***
presence of cachexia	logistic	1.07	1.02	1.11	0.00145	***
		Coeff	95% CI		p-value	
appetite (SNAQ)	linear	-0.10	-0.14	-0.06	2.43E-06	***
body weight loss (%)	linear	0.26	0.18	0.35	6.44E-09	***
ECOG performance status	linear	0.03	0.02	0.04	1.11E-06	***
QLQ-C30 function	linear	-0.55	-0.85	-0.25	3.43E-04	***
QLQ-C30 symptoms	linear	0.78	0.51	1.05	3.94E-08	***
QLQ-C30 quality of life	linear	-0.70	-1.07	-0.34	1.81E-04	***

**(B) LBP predictive value adjusted for age, sex, cancer type, cancer stage**						
Outcome	regression model	OR	95% CI		p-value	
occurrence of death	logistic	1.08	1.00	1.17	0.0395	*
presence of anorexia	logistic	1.13	1.06	1.20	8.14E-05	***
presence of cachexia	logistic	1.05	1.00	1.11	0.0266	*
		Coeff	95% CI		p-value	
appetite (SNAQ)	linear	-0.12	-0.16	-0.07	4.77E-07	***
body weight loss (%)	linear	0.26	0.16	0.36	1.48E-06	***
ECOG performance status	linear	0.03	0.02	0.04	2.34E-06	***
QLQ-C30 function	linear	-0.61	-0.94	-0.27	5.06E-04	***
QLQ-C30 symptoms	linear	0.81	0.52	1.10	1.97E-07	***
QLQ-C30 quality of life	linear	-0.74	-1.15	-0.33	5.90E-04	***

Results obtained from regression models adjusted for sex, age and cancer type (A) and for sex, age, cancer type and cancer stage (B). Regression coefficients represent the mean change in the response variable for one unit of change in the predictor variable while holding other predictors in the model constant.
For more information, please see the material and methods section as well as Loumaye et al, J Clin Endocrinol Metab 2015.
