# Supplementary material for: Increased gut permeability in cancer cachexia: mechanisms and clinical relevance
Source: Oncotarget. 2018 Apr 6;9(26):18224–38. doi: 10.18632/oncotarget.24804 (PMC5915068; doi:10.18632/oncotarget.24804)
Supplement: Supplementary file 2 [file oncotarget-09-18224-s002.docx]

**Table S2: Taxa and OTUs significantly affected by C26 cancer cell presence and/or the injection of antibody targeting IL-6.**

|  |  | **Mean** | | | **SEM** | | | **ANOVA** | | **Tukey post-tests** | | |
| --- | --- | --- | --- | --- | --- | --- | --- | --- | --- | --- | --- | --- |
| **Taxa** |  | **CT** | **C26** | **Anti-IL6** | **CT** | **C26** | **Anti-IL6** | **p-value** | **q-value** | **p-value** | **p-value** | **p-value** |
| **(in % of total sequences)** | |  |  |  |  |  |  |  |  | **C26/antiIL6** | **CT/antiIL6** | **CT/C26** |
| Enterococcaceae (family) | | 0.023 | 0.130 | 0.037 | 0.006 | 0.030 | 0.007 | 0.001 | 0.011 | 0.004 | 0.854 | 0.001 |
| Enterococcus (genus) | | 0.023 | 0.129 | 0.037 | 0.006 | 0.029 | 0.007 | 0.001 | 0.011 | 0.004 | 0.851 | 0.001 |
| unclassified_Firmicutes | | 4.319 | 0.650 | 1.957 | 0.766 | 0.164 | 0.580 | 0.001 | 0.011 | 0.251 | 0.019 | 0.000 |
| Verrucomicrobia (phylum) | | 3.749 | 19.440 | 7.929 | 1.018 | 3.296 | 2.592 | 0.001 | 0.011 | 0.010 | 0.474 | 0.001 |
| Verrucomicrobiae (class) | | 3.749 | 19.440 | 7.929 | 1.018 | 3.296 | 2.592 | 0.001 | 0.011 | 0.010 | 0.474 | 0.001 |
| Verrucomicrobiales (order) | | 3.749 | 19.440 | 7.929 | 1.018 | 3.296 | 2.592 | 0.001 | 0.011 | 0.010 | 0.474 | 0.001 |
| Verrucomicrobiaceae (family) | | 3.749 | 19.440 | 7.929 | 1.018 | 3.296 | 2.592 | 0.001 | 0.011 | 0.010 | 0.474 | 0.001 |
| Akkermansia (genus) | | 3.749 | 19.440 | 7.929 | 1.018 | 3.296 | 2.592 | 0.001 | 0.011 | 0.010 | 0.474 | 0.001 |
| unclassified_Bacteria | | 2.076 | 0.240 | 0.776 | 0.397 | 0.060 | 0.281 | 0.001 | 0.011 | 0.389 | 0.010 | 0.000 |
| Flavonifractor (genus) | | 0.556 | 2.300 | 1.716 | 0.073 | 0.282 | 0.423 | 0.002 | 0.020 | 0.362 | 0.030 | 0.001 |
| unclassified_Ruminococcaceae | | 13.360 | 7.369 | 16.866 | 1.174 | 1.462 | 2.229 | 0.002 | 0.029 | 0.002 | 0.323 | 0.050 |
| Clostridium XlVa (genus) | | 0.312 | 0.778 | 0.385 | 0.043 | 0.145 | 0.079 | 0.006 | 0.071 | 0.026 | 0.862 | 0.008 |
| Ruminococcaceae (family) | | 14.949 | 9.991 | 19.283 | 1.285 | 1.649 | 2.442 | 0.007 | 0.074 | 0.005 | 0.247 | 0.167 |
| Bacteroidaceae (family) | | 0.001 | 0.002 | 0.024 | 0.001 | 0.001 | 0.010 | 0.010 | 0.089 | 0.024 | 0.018 | 0.989 |
| Bacteroides (genus) | | 0.001 | 0.002 | 0.024 | 0.001 | 0.001 | 0.010 | 0.010 | 0.089 | 0.024 | 0.018 | 0.989 |
| Oscillibacter (genus) | | 0.900 | 0.246 | 0.625 | 0.232 | 0.054 | 0.109 | 0.020 | 0.168 | 0.203 | 0.418 | 0.016 |
| Acidaminococcaceae (family) | | 0.000 | 0.000 | 0.002 | 0.000 | 0.000 | 0.001 | 0.030 | 0.218 | 0.052 | 0.052 | 1.000 |
| Phascolarctobacterium (genus) | | 0.000 | 0.000 | 0.002 | 0.000 | 0.000 | 0.001 | 0.030 | 0.218 | 0.052 | 0.052 | 1.000 |
| Firmicutes (phylum) | | 78.179 | 55.388 | 74.090 | 2.825 | 6.800 | 7.502 | 0.034 | 0.223 | 0.098 | 0.883 | 0.038 |
| unclassified_Clostridiales | | 13.114 | 6.866 | 12.034 | 0.929 | 1.325 | 2.398 | 0.034 | 0.223 | 0.097 | 0.892 | 0.039 |
|  |  |  |  |  |  |  |  |  |  |  |  |  |
| **OTUID** | **Assignment using RDP seqmatch** |  |  |  |  |  |  |  |  |  |  |  |
| **In % of total sequences** | |  |  |  |  |  |  |  |  |  |  |  |
| OTU_34 | unclassified_Lachnospiraceae (1) | 0.303 | 0.099 | 0.106 | 0.037 | 0.016 | 0.023 | 0.000 | 0.002 | 0.984 | 0.000 | 0.000 |
| OTU_8 | unclassified_Lachnospiraceae (1) | 6.302 | 0.312 | 1.299 | 1.310 | 0.147 | 0.205 | 0.000 | 0.002 | 0.642 | 0.000 | 0.000 |
| OTU_42 | unclassified_Lachnospiraceae (0.93) | 0.081 | 0.010 | 0.034 | 0.012 | 0.005 | 0.012 | 0.000 | 0.011 | 0.260 | 0.012 | 0.000 |
| OTU_43 | unclassified_Lachnospiraceae (1) | 0.014 | 0.076 | 0.004 | 0.003 | 0.019 | 0.002 | 0.000 | 0.011 | 0.001 | 0.830 | 0.002 |
| OTU_4 | Akkermansia muciniphila (1) | 3.749 | 19.440 | 7.929 | 1.018 | 3.296 | 2.592 | 0.001 | 0.014 | 0.010 | 0.474 | 0.001 |
| OTU_41 | Enterococcus sp. (1) | 0.023 | 0.130 | 0.037 | 0.006 | 0.030 | 0.007 | 0.001 | 0.014 | 0.004 | 0.854 | 0.001 |
| OTU_3 | Oscillibacter sp. (1) | 12.910 | 5.749 | 15.838 | 1.174 | 1.287 | 2.369 | 0.001 | 0.019 | 0.001 | 0.455 | 0.019 |
| OTU_10 | Flavonifractor plautii (1) | 0.559 | 2.316 | 1.728 | 0.073 | 0.283 | 0.424 | 0.001 | 0.020 | 0.360 | 0.029 | 0.001 |
| OTU_87 | unclassified_Ruminococcaceae (0.98) | 0.154 | 0.988 | 0.698 | 0.036 | 0.134 | 0.218 | 0.003 | 0.031 | 0.373 | 0.045 | 0.002 |
| OTU_32 | Clostridium XIVa sp. (1) | 0.100 | 0.229 | 0.120 | 0.013 | 0.044 | 0.006 | 0.006 | 0.063 | 0.024 | 0.866 | 0.008 |
| OTU_23 | Clostridium sp. ASF356 (1) | 0.097 | 0.394 | 0.458 | 0.013 | 0.099 | 0.089 | 0.007 | 0.073 | 0.827 | 0.009 | 0.033 |
| OTU_63 | Bacteroides eggerthii (1) | 0.000 | 0.000 | 0.003 | 0.000 | 0.000 | 0.001 | 0.010 | 0.088 | 0.020 | 0.020 | 1.000 |
| OTU_13 | unclassified_Lachnospiraceae (0.95) | 2.123 | 0.252 | 0.785 | 0.592 | 0.062 | 0.401 | 0.013 | 0.107 | 0.641 | 0.081 | 0.012 |
| OTU_26 | Clostridium aldenense (1) | 0.194 | 0.481 | 0.231 | 0.032 | 0.104 | 0.066 | 0.023 | 0.180 | 0.063 | 0.933 | 0.030 |
| OTU_14 | unclassified_Lachnospiraceae (1) | 0.912 | 0.339 | 0.812 | 0.073 | 0.114 | 0.225 | 0.032 | 0.208 | 0.093 | 0.889 | 0.037 |
| OTU_21 | unclassified_Lachnospiraceae (0.90) | 0.610 | 0.436 | 1.152 | 0.118 | 0.085 | 0.286 | 0.032 | 0.208 | 0.032 | 0.121 | 0.787 |
| OTU_40 | Clostridium XIVa sp. (1) | 0.033 | 0.090 | 0.046 | 0.008 | 0.020 | 0.013 | 0.029 | 0.208 | 0.111 | 0.784 | 0.029 |
